# Supplementary figures and images for: Biocontrol potential of endophytic Pseudomonas strain IALR1619 against two Pythium species in cucumber and hydroponic lettuce
Source: PLoS One. 2024 Feb 26;19(2):e0298514. doi: 10.1371/journal.pone.0298514 (PMC10896519; doi:10.1371/journal.pone.0298514)

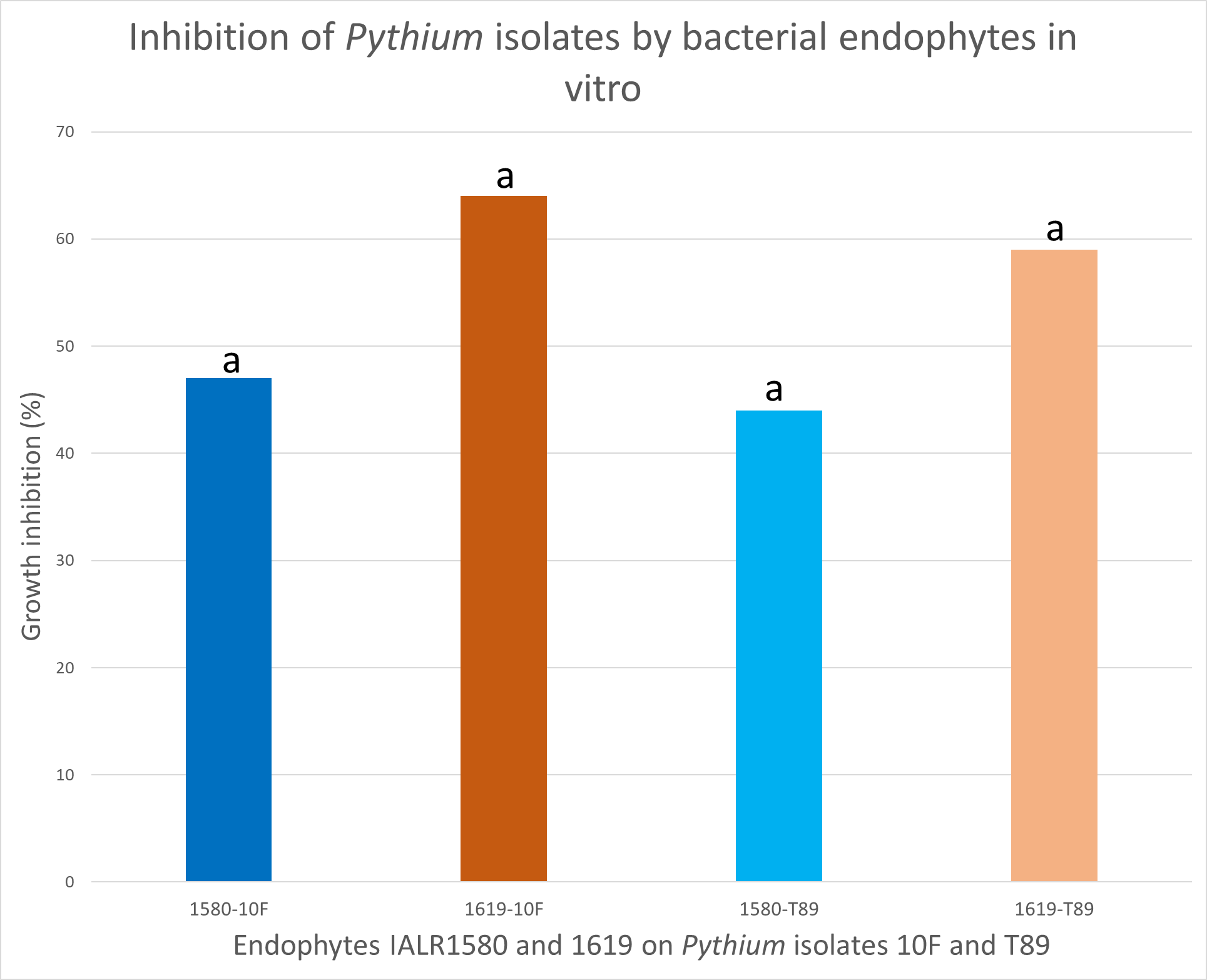

Supplement: S1 Fig — Growth inhibition bars having same letter above them are not significantly different at P = 0.05. (TIF) [file pone.0298514.s001.tif]
