# Supplementary material for: Biocontrol potential of endophytic Pseudomonas strain IALR1619 against two Pythium species in cucumber and hydroponic lettuce
Source: PLoS One. 2024 Feb 26;19(2):e0298514. doi: 10.1371/journal.pone.0298514 (PMC10896519; doi:10.1371/journal.pone.0298514)
Supplement: S1 Text — (Tables 1 and 2) Inhibition percentages of Pythium 10F and T89 isolates in the presence of endophytes IALR1580 and 1619. (Table 3) Statistical analysis for inhibition of Pythium isolates by IALR1580 and 1619 endophytes. (RTF) [file pone.0298514.s002.rtf]

observation number	Endophyte_Pythium	Inhibition_pct	
1	IALR1580-10F	47.682119205	
2	IALR1580-10F	47.682119205	
3	IALR1580-10F	46.357615894	
4	IALR1580-T89	44.444444444	
5	IALR1580-T90	44.444444444	
6	IALR1580-T91	43.055555556	
7	IALR1619-10F	64.900662252	
8	IALR1619-10F	63.57615894	
9	IALR1619-10F	64.238410596	
10	IALR1619-T89	57.638888889	
11	IALR1619-T90	59.027777778	
12	IALR1619-T91	60.416666667	
N = 12	

Analysis Variable : Inhibition_pct	
N Obs	N	Mean	Std Dev	Minimum	Maximum	
12	12	53.62	8.71	43.06	64.90	


Analysis Variable : Inhibition_pct	
Endophyte_Pythium	N Obs	N	Mean	Std Dev	Minimum	Maximum	
IALR1580-10F	3	3	47.24	0.76	46.36	47.68	
IALR1580-T89	3	3	43.98	0.80	43.06	44.44	
IALR1619-10F	3	3	64.24	0.66	63.58	64.90	
IALR1619-T89	3	3	59.03	1.39	57.64	60.42	


Wilcoxon Scores (Rank Sums) for Variable Inhibition_pct
Classified by Variable Endophyte_Pythium	
Endophyte_Pythium	N	Sum of
Scores	Expected
Under H0	Std Dev
Under H0	Mean
Score	
IALR1580-10F	3	15.0	19.50	5.398864	5.0	
IALR1580-T89	3	6.0	19.50	5.398864	2.0	
IALR1619-10F	3	33.0	19.50	5.398864	11.0	
IALR1619-T89	3	24.0	19.50	5.398864	8.0	
Average scores were used for ties.	


Kruskal-Wallis Test	
Chi-Square	DF	Pr > ChiSq	
10.4211	3	0.0153	


Pairwise Two-Sided Multiple Comparison Analysis	
Dwass, Steel, Critchlow-Fligner Method	
Variable: Inhibition_pct	
Endophyte_Pythium	Wilcoxon Z	DSCF Value	Pr > DSCF	
IALR1580-10F vs. IALR1580-T89	1.9926	2.8180	0.1907	
IALR1580-10F vs. IALR1619-10F	-1.9640	2.7775	0.2017	
IALR1580-10F vs. IALR1619-T89	-1.9640	2.7775	0.2017	
IALR1580-T89 vs. IALR1619-10F	-1.9926	2.8180	0.1907	
IALR1580-T89 vs. IALR1619-T89	-1.9926	2.8180	0.1907	
IALR1619-10F vs. IALR1619-T89	1.9640	2.7775	0.2017	
