# Supplementary material for: Biocontrol potential of endophytic Pseudomonas strain IALR1619 against two Pythium species in cucumber and hydroponic lettuce
Source: PLoS One. 2024 Feb 26;19(2):e0298514. doi: 10.1371/journal.pone.0298514 (PMC10896519; doi:10.1371/journal.pone.0298514)
Supplement: S2 Text — (Table 1) Shoot dry weight of Cucumber plants treated with and without endophyte IALR1619. (Table 2) Dry shoot weight comparison for IALR1619 inoculated and non-inoculated cucumber plants. (Tables 3 and 4) Chi Square test to ascertain the effect of IALR1619 on cucumber seedlings inoculated with Pythium. (RTF) [file pone.0298514.s003.rtf]

Obs	Plant	Treatment	ShootDryWt_g	
1	1	NegCon	0.332	
2	2	NegCon	0.148	
3	3	NegCon	0.233	
4	4	NegCon	0.244	
5	5	NegCon	0.264	
6	6	NegCon	0.171	
7	7	NegCon	0.267	
8	8	NegCon	0.274	
9	9	NegCon	0.273	
10	10	NegCon	0.305	
11	11	NegCon	0.247	
12	12	NegCon	0.254	
13	13	NegCon	0.285	
14	14	NegCon	0.293	
15	15	NegCon	0.268	
16	16	NegCon	0.156	
17	17	NegCon	0.229	
18	18	NegCon	0.258	
19	19	NegCon	0.318	
20	20	NegCon	0.305	
21	21	NegCon	0.302	
22	1	IALR1619	0.208	
23	2	IALR1619	0.265	
24	3	IALR1619	0.23	
25	4	IALR1619	0.26	
26	5	IALR1619	0.289	
27	6	IALR1619	0.304	
28	7	IALR1619	0.293	
29	8	IALR1619	0.252	
30	9	IALR1619	0.287	
31	10	IALR1619	0.278	
32	11	IALR1619	0.292	
33	12	IALR1619	0.287	
34	13	IALR1619	0.329	
35	14	IALR1619	0.317	
36	15	IALR1619	0.31	
37	16	IALR1619	0.265	
38	17	IALR1619	0.208	
39	18	IALR1619	0.227	
40	19	IALR1619	0.22	
41	20	IALR1619	0.226	
42	21	IALR1619	0.27	

Treatment	Method	N	Mean	Std Dev	Std Err	Minimum	Maximum	
1619only		21	0.2675	0.0363	0.00792	0.2080	0.3290	
NegCon		21	0.2584	0.0500	0.0109	0.1480	0.3320	
Diff (1-2)	Pooled		0.00910	0.0437	0.0135			
Diff (1-2)	Satterthwaite		0.00910		0.0135			


Treatment	Method	Mean	95% CL Mean	Std Dev	95% CL Std Dev	
1619only		0.2675	0.2509	0.2840	0.0363	0.0278	0.0524	
NegCon		0.2584	0.2356	0.2811	0.0500	0.0382	0.0722	
Diff (1-2)	Pooled	0.00910	-0.0136	Infty	0.0437	0.0359	0.0559	
Diff (1-2)	Satterthwaite	0.00910	-0.0137	Infty				


Method	Variances	DF	t Value	Pr > t	
Pooled	Equal	40	0.67	0.2519	
Satterthwaite	Unequal	36.516	0.67	0.2521	


Equality of Variances	
Method	Num DF	Den DF	F Value	Pr > F	
Folded F	20	20	1.89	0.1619	


Table of Treatment by Survivability	
Treatment(Treatment)	Survivability(Survivability)	
Frequency
Expected
Cell Chi-Square
Row Pct
Col Pct	Dead	Live	Total	
1619andPy	9
13.5
1.5
40.91
33.33	13
8.5
2.3824
59.09
76.47	22


	
PosCont	18
13.5
1.5
81.82
66.67	4
8.5
2.3824
18.18
23.53	22


	
Total	27	17	44	


Statistics for Table of Treatment by Survivability	


Statistic	DF	Value	Prob	
Chi-Square	1	7.7647	0.0053	
Likelihood Ratio Chi-Square	1	8.0750	0.0045	
Continuity Adj. Chi-Square	1	6.1351	0.0133	
Mantel-Haenszel Chi-Square	1	7.5882	0.0059	
Phi Coefficient		-0.4201		
Contingency Coefficient		0.3873		
Cramer's V		-0.4201		


Fisher's Exact Test	
Cell (1,1) Frequency (F)	9	
Left-sided Pr <= F	0.0061	
Right-sided Pr >= F	0.9992	
		
Table Probability (P)	0.0053	
Two-sided Pr <= P	0.0122	

Sample Size = 44	

Table of Treatment by Survivability	
Treatment(Treatment)	Survivability(Survivability)	
Frequency
Expected
Cell Chi-Square
Percent
Row Pct
Col Pct	Dead	Live	Total	
1619andPy	7
12.273
2.2653
12.73
25.93
28.00	20
14.727
1.8878
36.36
74.07
66.67	27


49.09

	
PosCon	18
12.727
2.1844
32.73
64.29
72.00	10
15.273
1.8203
18.18
35.71
33.33	28


50.91

	
Total	25
45.45	30
54.55	55
100.00	


Statistics for Table of Treatment by Survivability	


Statistic	DF	Value	Prob	
Chi-Square	1	8.1578	0.0043	
Likelihood Ratio Chi-Square	1	8.3895	0.0038	
Continuity Adj. Chi-Square	1	6.6840	0.0097	
Mantel-Haenszel Chi-Square	1	8.0095	0.0047	
Phi Coefficient		-0.3851		
Contingency Coefficient		0.3594		
Cramer's V		-0.3851		


Fisher's Exact Test	
Cell (1,1) Frequency (F)	7	
Left-sided Pr <= F	0.0045	
Right-sided Pr >= F	0.9992	
		
Table Probability (P)	0.0038	
Two-sided Pr <= P	0.0066	

Sample Size = 55	
