# Supplementary material for: Biocontrol potential of endophytic Pseudomonas strain IALR1619 against two Pythium species in cucumber and hydroponic lettuce
Source: PLoS One. 2024 Feb 26;19(2):e0298514. doi: 10.1371/journal.pone.0298514 (PMC10896519; doi:10.1371/journal.pone.0298514)
Supplement: S3 Text — (RTF) [file pone.0298514.s004.rtf]

Obs	TRT	Cultivar	Block	FrShootWt	DryShootWt	FreshRoot	DryRoot	Ht_cm	
1	Pos Control	Cristabel	1	35.56	0.968	6.35	0.635	.	
2	Pos Control	Cristabel	1	20.49	1.242	6.75	0.516	.	
3	Pos Control	Cristabel	1	25.65	1.692	6.1	0.668	.	
4	Pos Control	Cristabel	1	26.46	1.427	5.62	0.635	.	
5	Pos Control	Cristabel	1	22.01	1.155	6.91	0.552	.	
6	Pos Control	Cristabel	1	23.09	1.22	5.37	0.599	.	
7	Pos Control	Cristabel	1	23.07	0.916	5.02	0.53	.	
8	Pos Control	Cristabel	1	26.36	1.063	5.22	0.529	.	
9	Pos Control	Cristabel	1	17.91	1.439	7.32	0.584	.	
10	Pos Control	Cristabel	2	26.48	1.248	4.93	0.429	.	
11	Pos Control	Cristabel	2	22.55	1.265	5.34	0.487	.	
12	Pos Control	Cristabel	2	23.1	1.042	5.63	0.452	.	
13	Pos Control	Cristabel	2	16.22	1.189	5.86	0.458	.	
14	Pos Control	Cristabel	2	26.01	1.426	5.35	0.462	.	
15	Pos Control	Cristabel	2	21.71	1.133	6.21	0.46	.	
16	Pos Control	Cristabel	2	26.07	0.825	5.06	0.446	.	
17	Pos Control	Cristabel	2	18.52	1.002	5.97	0.471	.	
18	Pos Control	Cristabel	2	25.35	1.33	5.61	0.45	.	
19	Pos Control	Cristabel	3	23.27	0.839	6.02	0.436	.	
20	Pos Control	Cristabel	3	24.58	1.152	4.8	0.392	.	
21	Pos Control	Cristabel	3	19.14	0.638	5.11	0.41	.	
22	Pos Control	Cristabel	3	17.07	1.129	4.98	0.42	.	
23	Pos Control	Cristabel	3	17	0.879	6.06	0.445	.	
24	Pos Control	Cristabel	3	13.5	0.922	5.03	0.433	.	
25	Pos Control	Cristabel	3	16.52	1.007	4.79	0.423	.	
26	Neg Control	Cristabel	1	26.23	1.34	5.86	0.522	.	
27	Neg Control	Cristabel	1	29.63	1.317	5.24	0.532	.	
28	Neg Control	Cristabel	1	22.44	1.123	5.03	0.404	.	
29	Neg Control	Cristabel	1	27.47	1.247	5.1	0.449	.	
30	Neg Control	Cristabel	1	22.72	1.751	5.34	0.439	.	
31	Neg Control	Cristabel	1	27.32	1.621	4.99	0.428	.	
32	Neg Control	Cristabel	1	32.33	1.472	5.49	0.448	.	
33	Neg Control	Cristabel	1	29.99	1.26	8.12	0.477	.	
34	Neg Control	Cristabel	1	36.37	1.662	6.55	0.421	.	
35	Neg Control	Cristabel	2	30.29	1.468	6.89	0.411	.	
36	Neg Control	Cristabel	2	30.78	1.573	7.22	0.429	.	
37	Neg Control	Cristabel	2	28.18	1.429	5.02	0.422	.	
38	Neg Control	Cristabel	2	27.54	1.284	5.48	0.392	.	
39	Neg Control	Cristabel	2	38.58	1.511	9.86	0.551	.	
40	Neg Control	Cristabel	2	27.01	1.365	4.75	0.426	.	
41	Neg Control	Cristabel	2	27.62	1.48	5.21	0.402	.	
42	Neg Control	Cristabel	2	34.62	1.588	5.5	0.511	.	
43	Neg Control	Cristabel	2	32.36	1.782	5.02	0.457	.	
44	Neg Control	Cristabel	3	19.13	1.065	5.22	0.457	.	
45	Neg Control	Cristabel	3	25.12	0.857	6.26	0.434	.	
46	Neg Control	Cristabel	3	30.33	1.37	4.79	0.387	.	
47	Neg Control	Cristabel	3	22.7	1.275	4.6	0.512	.	
48	Neg Control	Cristabel	3	13.28	1.214	5.45	0.427	.	
49	Neg Control	Cristabel	3	29.2	1.222	7.79	0.445	.	
50	Neg Control	Cristabel	3	27.08	0.712	5.51	0.406	.	
51	Neg Control	Cristabel	3	26.12	1.348	5.22	0.394	.	
52	Neg Control	Cristabel	3	24.09	1.233	6.59	0.425	.	
53	IALR1619	Cristabel	1	29.66	1.607	5.77	0.544	.	
54	IALR1619	Cristabel	1	27.4	1.171	4.58	0.472	.	
55	IALR1619	Cristabel	1	24.21	1.235	6.01	0.498	.	
56	IALR1619	Cristabel	1	17.87	1.271	7.74	0.463	.	
57	IALR1619	Cristabel	1	24.26	0.902	6.38	0.538	.	
58	IALR1619	Cristabel	1	31.25	1.568	5.83	0.453	.	
59	IALR1619	Cristabel	1	28.87	1.335	6.1	0.452	.	
60	IALR1619	Cristabel	1	26.78	1.429	6.63	0.425	.	
61	IALR1619	Cristabel	1	24.23	1.429	6.66	0.436	.	
62	IALR1619	Cristabel	2	36.66	1.322	5.7	0.498	.	
63	IALR1619	Cristabel	2	35.14	1.105	5.25	0.503	.	
64	IALR1619	Cristabel	2	28.12	1.749	7.46	0.446	.	
65	IALR1619	Cristabel	2	30.49	1.348	5.18	0.588	.	
66	IALR1619	Cristabel	2	23.5	1.109	6.5	0.526	.	
67	IALR1619	Cristabel	2	29.64	1.785	4.8	0.606	.	
68	IALR1619	Cristabel	2	23.44	1.761	7.02	0.478	.	
69	IALR1619	Cristabel	2	28.8	1.367	7.38	0.493	.	
70	IALR1619	Cristabel	2	34.79	1.404	6.21	0.625	.	
71	IALR1619	Cristabel	3	24.6	1.485	5.17	0.608	.	
72	IALR1619	Cristabel	3	25.11	1.384	7.95	0.505	.	
73	IALR1619	Cristabel	3	39.07	1.649	6.71	0.581	.	
74	IALR1619	Cristabel	3	25.83	1.883	7.43	0.576	.	
75	IALR1619	Cristabel	3	27.42	1.578	5.27	0.5	.	
76	IALR1619	Cristabel	3	31.52	1.288	5.92	0.511	.	
77	IALR1619	Cristabel	3	32.91	2	5.84	0.582	.	
78	IALR1619	Cristabel	3	29.23	1.25	5.2	0.709	.	
79	IALR1619	Cristabel	3	36.51	1.384	7.72	0.475	.	
80	IALR1580	Cristabel	1	19.38	0.789	5.47	0.503	.	
81	IALR1580	Cristabel	1	22.17	1.21	6.56	0.435	.	
82	IALR1580	Cristabel	1	23.59	1.101	5.52	0.511	.	
83	IALR1580	Cristabel	1	19.92	1.108	6.1	0.503	.	
84	IALR1580	Cristabel	1	16.56	1.108	5.32	0.513	.	
85	IALR1580	Cristabel	1	14.38	1.37	5.23	0.492	.	
86	IALR1580	Cristabel	1	18.79	1.166	5.42	0.519	.	
87	IALR1580	Cristabel	1	22.551	1.095	4.48	0.52	.	
88	IALR1580	Cristabel	1	20.37	1.191	5.25	0.523	.	
89	IALR1580	Cristabel	2	20.57	1.185	4.96	0.506	.	
90	IALR1580	Cristabel	2	13.18	1.047	4.76	0.483	.	
91	IALR1580	Cristabel	2	21.04	1.126	4.27	0.447	.	
92	IALR1580	Cristabel	2	19.86	0.75	6.42	0.51	.	
93	IALR1580	Cristabel	2	22	1.109	5.72	0.471	.	
94	IALR1580	Cristabel	2	23.64	1.208	4.16	0.405	.	
95	IALR1580	Cristabel	2	15.34	1.118	5.46	0.407	.	
96	IALR1580	Cristabel	2	21.92	1.061	5.06	0.398	.	
97	IALR1580	Cristabel	2	20.71	0.655	5.24	0.543	.	
98	IALR1580	Cristabel	3	14.27	0.846	4.63	0.443	.	
99	IALR1580	Cristabel	3	15.62	1.108	4.02	0.411	.	
100	IALR1580	Cristabel	3	13.12	0.782	3.96	0.414	.	
101	IALR1580	Cristabel	3	20.96	1.495	5.69	0.44	.	
102	IALR1580	Cristabel	3	16.2	0.879	3.32	0.407	.	
103	IALR1580	Cristabel	3	13.9	0.798	4.34	0.401	.	
104	IALR1580	Cristabel	3	30.49	0.751	4.2	0.515	.	
105	Pos Control	RomaineRedRosie	1	12.24	0.802	5.38	0.472	18.5	
106	Pos Control	RomaineRedRosie	1	12.23	0.754	5.02	0.46	18.5	
107	Pos Control	RomaineRedRosie	1	10.26	1.059	5.18	0.448	20.7	
108	Pos Control	RomaineRedRosie	1	12.03	0.846	5.37	0.472	18.7	
109	Pos Control	RomaineRedRosie	1	16.01	0.841	4.57	0.454	20.3	
110	Pos Control	RomaineRedRosie	1	12.3	0.851	5.65	0.44	19.1	
111	Pos Control	RomaineRedRosie	1	11.43	0.682	6.15	0.46	19.2	
112	Pos Control	RomaineRedRosie	1	10.72	0.739	5.77	0.43	18.8	
113	Pos Control	RomaineRedRosie	1	10.12	0.683	5.88	0.451	19.5	
114	Pos Control	RomaineRedRosie	2	27.13	1.427	4.59	0.45	26	
115	Pos Control	RomaineRedRosie	2	21.15	1.69	6.17	0.549	25.2	
116	Pos Control	RomaineRedRosie	2	27.67	1.861	6.34	0.466	24.5	
117	Pos Control	RomaineRedRosie	2	24.78	1.006	6.41	0.614	24	
118	Pos Control	RomaineRedRosie	2	17.73	0.875	7.35	0.547	25.5	
119	Pos Control	RomaineRedRosie	2	22.33	1.296	7.26	0.566	25.7	
120	Pos Control	RomaineRedRosie	2	32.06	1.614	6.89	0.518	27.5	
121	Pos Control	RomaineRedRosie	2	19.92	1.155	4.76	0.446	25.7	
122	Pos Control	RomaineRedRosie	2	14.91	1.319	5.38	0.475	22.8	
123	Pos Control	RomaineRedRosie	3	12.96	1.302	6.1	0.438	23.7	
124	Pos Control	RomaineRedRosie	3	16.34	0.841	6.71	0.425	21.1	
125	Pos Control	RomaineRedRosie	3	20.73	0.779	5.02	0.471	24	
126	Pos Control	RomaineRedRosie	3	13.7	1.135	6.04	0.444	21.7	
127	Pos Control	RomaineRedRosie	3	13.11	1.332	6.73	0.498	21.5	
128	Pos Control	RomaineRedRosie	3	13.4	0.802	4.61	0.516	23	
129	Pos Control	RomaineRedRosie	3	19.61	1.249	5.12	0.498	22.6	
130	Pos Control	RomaineRedRosie	3	22.72	1.031	5.66	0.505	24.4	
131	Pos Control	RomaineRedRosie	3	20.29	0.858	5.81	0.395	23.5	
132	Neg Control	RomaineRedRosie	1	37.34	1.833	6.9	0.561	24.6	
133	Neg Control	RomaineRedRosie	1	27.86	1.939	5.86	0.652	25	
134	Neg Control	RomaineRedRosie	1	31.09	1.806	7.67	0.519	25.5	
135	Neg Control	RomaineRedRosie	1	37.32	2.45	6.27	0.542	26.5	
136	Neg Control	RomaineRedRosie	1	31.86	1.636	5.38	0.538	25.5	
137	Neg Control	RomaineRedRosie	1	29.99	1.568	6.63	0.515	26	
138	Neg Control	RomaineRedRosie	1	26.9	2.025	6.5	0.545	27.5	
139	Neg Control	RomaineRedRosie	1	27.67	2.122	8.08	0.576	25.7	
140	Neg Control	RomaineRedRosie	1	34.38	1.799	5.77	0.556	25	
141	Neg Control	RomaineRedRosie	2	49.59	1.595	7.45	0.62	25.5	
142	Neg Control	RomaineRedRosie	2	29.33	2.484	6.73	0.495	24.1	
143	Neg Control	RomaineRedRosie	2	25.39	1.349	5.37	0.74	24.7	
144	Neg Control	RomaineRedRosie	2	23.56	2.706	6.06	0.65	24	
145	Neg Control	RomaineRedRosie	2	46.92	1.681	9.25	0.683	26.7	
146	Neg Control	RomaineRedRosie	2	39.08	2.772	6.48	0.452	24.1	
147	Neg Control	RomaineRedRosie	2	34.08	1.358	7.96	0.47	26.2	
148	Neg Control	RomaineRedRosie	2	30.87	1.642	9.2	0.533	26.7	
149	Neg Control	RomaineRedRosie	2	30.89	1.873	10.16	0.564	24.6	
150	Neg Control	RomaineRedRosie	3	39.5	1.714	6.31	0.603	24.7	
151	Neg Control	RomaineRedRosie	3	19.32	1.875	8.53	0.746	24.6	
152	Neg Control	RomaineRedRosie	3	27.14	1.557	5.51	0.532	25	
153	Neg Control	RomaineRedRosie	3	32.12	2.006	6.41	0.497	25.5	
154	Neg Control	RomaineRedRosie	3	43.23	2.216	8.14	0.612	24.6	
155	Neg Control	RomaineRedRosie	3	32	2.344	10.53	0.474	24.7	
156	Neg Control	RomaineRedRosie	3	35.85	2.643	8.64	0.63	25.7	
157	Neg Control	RomaineRedRosie	3	38.97	1.993	8.98	0.685	26.1	
158	Neg Control	RomaineRedRosie	3	31.98	1.104	5.63	0.457	25.2	
159	IALR1619	RomaineRedRosie	1	18.19	1.292	8.36	0.544	19.2	
160	IALR1619	RomaineRedRosie	1	24.54	1.531	6.27	0.472	23.9	
161	IALR1619	RomaineRedRosie	1	27.64	1.725	7.02	0.498	23.7	
162	IALR1619	RomaineRedRosie	1	29.64	2	7.15	0.463	23.9	
163	IALR1619	RomaineRedRosie	1	20.77	1.808	5.16	0.538	22	
164	IALR1619	RomaineRedRosie	1	18.84	1.58	8.51	0.453	21.5	
165	IALR1619	RomaineRedRosie	1	26.44	1.308	6.22	0.452	24	
166	IALR1619	RomaineRedRosie	1	24.46	1.414	8.72	0.425	23.5	
167	IALR1619	RomaineRedRosie	1	28.94	1.628	5.09	0.436	23.9	
168	IALR1619	RomaineRedRosie	2	25.72	1.537	6.92	0.461	20.7	
169	IALR1619	RomaineRedRosie	2	24.62	1.482	7.93	0.432	24.9	
170	IALR1619	RomaineRedRosie	2	15.78	1.659	7.88	0.438	23.1	
171	IALR1619	RomaineRedRosie	2	17.61	1.716	6.59	0.429	23	
172	IALR1619	RomaineRedRosie	2	15.09	1.484	4.82	0.533	23.5	
173	IALR1619	RomaineRedRosie	2	30.47	1.852	6.08	0.482	24	
174	IALR1619	RomaineRedRosie	2	26.01	0.949	5.66	0.497	23.6	
175	IALR1619	RomaineRedRosie	2	28.8	0.996	6.46	0.41	22.2	
176	IALR1619	RomaineRedRosie	2	28.53	0.905	5.97	0.52	24.7	
177	IALR1619	RomaineRedRosie	3	28.71	1.305	8.12	0.629	25.7	
178	IALR1619	RomaineRedRosie	3	23.42	2.135	7.11	0.527	25.1	
179	IALR1619	RomaineRedRosie	3	23.89	1.968	6.3	0.488	25.1	
180	IALR1619	RomaineRedRosie	3	36.32	1.769	6.14	0.492	24.9	
181	IALR1619	RomaineRedRosie	3	20.28	1.501	6.53	0.504	21.5	
182	IALR1619	RomaineRedRosie	3	34.69	1.304	7.09	0.53	24.8	
183	IALR1619	RomaineRedRosie	3	24.61	1.349	5.53	0.438	23.5	
184	IALR1619	RomaineRedRosie	3	21.94	1.358	5.92	0.468	23.5	
185	IALR1619	RomaineRedRosie	3	19.2	1.373	4.86	0.476	19.5	
186	IALR1580	RomaineRedRosie	1	21.41	1.524	4.55	0.566	20.5	
187	IALR1580	RomaineRedRosie	1	27.21	1.373	5.55	0.5	23.7	
188	IALR1580	RomaineRedRosie	1	23.39	0.995	5.03	0.527	23	
189	IALR1580	RomaineRedRosie	1	14.93	1.458	4.87	0.495	21.9	
190	IALR1580	RomaineRedRosie	1	20.62	1.468	5.84	0.559	23.7	
191	IALR1580	RomaineRedRosie	1	22.53	1.489	6.06	0.555	23.7	
192	IALR1580	RomaineRedRosie	1	22.86	1.765	5.57	0.581	24.2	
193	IALR1580	RomaineRedRosie	1	22.83	1.375	4.63	0.528	23.5	
194	IALR1580	RomaineRedRosie	1	21.9	1.446	5.27	0.506	19.9	
195	IALR1580	RomaineRedRosie	2	9.2	0.86	4.52	0.513	20.2	
196	IALR1580	RomaineRedRosie	2	11.21	0.8	5.12	0.501	19.7	
197	IALR1580	RomaineRedRosie	2	10.89	0.842	5.44	0.513	23.1	
198	IALR1580	RomaineRedRosie	2	13.74	1.007	4.93	0.54	23.3	
199	IALR1580	RomaineRedRosie	2	12.96	0.69	5.41	0.502	24.6	
200	IALR1580	RomaineRedRosie	2	14.02	0.667	4.44	0.518	24.5	
201	IALR1580	RomaineRedRosie	2	16.96	0.85	4.9	0.445	25.5	
202	IALR1580	RomaineRedRosie	2	11.11	0.728	5.3	0.486	22.1	
203	IALR1580	RomaineRedRosie	2	13.69	0.635	4.76	0.502	23.1	
204	IALR1580	RomaineRedRosie	3	22.48	1.271	4.94	0.591	25.5	
205	IALR1580	RomaineRedRosie	3	27.56	1.618	6.89	0.439	26.1	
206	IALR1580	RomaineRedRosie	3	23.1	1.086	6.17	0.478	24.2	
207	IALR1580	RomaineRedRosie	3	18.34	1.47	5.08	0.451	25.7	
208	IALR1580	RomaineRedRosie	3	15.41	1.47	4.28	0.516	24	
209	IALR1580	RomaineRedRosie	3	18.99	0.786	3.94	0.419	24.6	
210	IALR1580	RomaineRedRosie	3	14.74	0.95	4.82	0.54	22.1	
211	IALR1580	RomaineRedRosie	3	27.4	0.959	3.94	0.654	27.2	
212	IALR1580	RomaineRedRosie	3	12.1	1.597	5.8	0.474	22.2	

Class Level Information	
Class	Levels	Values	
TRT	4	IALR1580 IALR1619 Neg Control Pos Control	
Block	3	1 2 3	


Number of Observations Read	104	
Number of Observations Used	104	

Source	DF	Sum of Squares	Mean Square	F Value	Pr > F	
Model	5	1753.226724	350.645345	16.30	<.0001	
Error	98	2108.227633	21.512527			
Corrected Total	103	3861.454358				


R-Square	Coeff Var	Root MSE	FrShootWt Mean	
0.454033	18.81268	4.638160	24.65443	


Source	DF	Type I SS	Mean Square	F Value	Pr > F	
TRT	3	1591.583594	530.527865	24.66	<.0001	
Block	2	161.643131	80.821565	3.76	0.0268	


Source	DF	Type III SS	Mean Square	F Value	Pr > F	
TRT	3	1638.141893	546.047298	25.38	<.0001	
Block	2	161.643131	80.821565	3.76	0.0268	


TRT	FrShootWt LSMEAN	LSMEAN Number	
IALR1580	19.0897781	1	
IALR1619	28.7892593	2	
Neg Control	27.7233333	3	
Pos Control	22.1761381	4	


Least Squares Means for effect TRT
Pr > |t| for H0: LSMean(i)=LSMean(j)

Dependent Variable: FrShootWt	
i/j	1	2	3	4	
1		<.0001	<.0001	0.0934	
2	<.0001		0.8331	<.0001	
3	<.0001	0.8331		0.0002	
4	0.0934	<.0001	0.0002		


Class Level Information	
Class	Levels	Values	
TRT	4	IALR1580 IALR1619 Neg Control Pos Control	
Block	3	1 2 3	


Number of Observations Read	108	
Number of Observations Used	106	

Source	DF	Sum of Squares	Mean Square	F Value	Pr > F	
Model	5	4373.451219	874.690244	25.63	<.0001	
Error	100	3412.880422	34.128804			
Corrected Total	105	7786.331642				


R-Square	Coeff Var	Root MSE	FrShootWt Mean	
0.561683	25.00345	5.841986	23.36472	


Source	DF	Type I SS	Mean Square	F Value	Pr > F	
TRT	3	4335.052671	1445.017557	42.34	<.0001	
Block	2	38.398548	19.199274	0.56	0.5715	


Source	DF	Type III SS	Mean Square	F Value	Pr > F	
TRT	3	4344.715904	1448.238635	42.43	<.0001	
Block	2	38.398548	19.199274	0.56	0.5715	


TRT	FrShootWt LSMEAN	LSMEAN Number	
IALR1580	18.2066667	1	
IALR1619	24.6351852	2	
Neg Control	33.1196296	3	
Pos Control	16.9951777	4	


Least Squares Means for effect TRT
Pr > |t| for H0: LSMean(i)=LSMean(j)

Dependent Variable: FrShootWt	
i/j	1	2	3	4	
1		0.0006	<.0001	0.8777	
2	0.0006		<.0001	<.0001	
3	<.0001	<.0001		<.0001	
4	0.8777	<.0001	<.0001		


Class Level Information	
Class	Levels	Values	
TRT	4	IALR1580 IALR1619 Neg Control Pos Control	
Block	3	1 2 3	


Number of Observations Read	108	
Number of Observations Used	108	

Source	DF	Sum of Squares	Mean Square	F Value	Pr > F	
Model	5	175.0657407	35.0131481	11.01	<.0001	
Error	102	324.3705556	3.1801035			
Corrected Total	107	499.4362963				


R-Square	Coeff Var	Root MSE	Ht_cm Mean	
0.350527	7.551550	1.783284	23.61481	


Source	DF	Type I SS	Mean Square	F Value	Pr > F	
TRT	3	121.4777778	40.4925926	12.73	<.0001	
Block	2	53.5879630	26.7939815	8.43	0.0004	


Source	DF	Type III SS	Mean Square	F Value	Pr > F	
TRT	3	121.4777778	40.4925926	12.73	<.0001	
Block	2	53.5879630	26.7939815	8.43	0.0004	


TRT	Ht_cm LSMEAN	LSMEAN Number	
IALR1580	23.4000000	1	
IALR1619	23.2925926	2	
Neg Control	25.3333333	3	
Pos Control	22.4333333	4	


Least Squares Means for effect TRT
Pr > |t| for H0: LSMean(i)=LSMean(j)

Dependent Variable: Ht_cm	
i/j	1	2	3	4	
1		0.9962	0.0007	0.1979	
2	0.9962		0.0003	0.2935	
3	0.0007	0.0003		<.0001	
4	0.1979	0.2935	<.0001		


Wilcoxon Scores (Rank Sums) for Variable DryShootWt
Classified by Variable TRT	
TRT	N	Sum of
Scores	Expected
Under H0	Std Dev
Under H0	Mean
Score	
Pos Control	25	980.50	1312.50	131.453610	39.220000	
Neg Control	27	1789.00	1417.50	134.870265	66.259259	
IALR1619	27	1962.00	1417.50	134.870265	72.666667	
IALR1580	25	728.50	1312.50	131.453610	29.140000	
Average scores were used for ties.	


Kruskal-Wallis Test	
Chi-Square	DF	Pr > ChiSq	
37.5229	3	<.0001	


Pairwise Two-Sided Multiple Comparison Analysis	
Dwass, Steel, Critchlow-Fligner Method	
Variable: DryShootWt	
TRT	Wilcoxon Z	DSCF Value	Pr > DSCF	
Pos Control vs. Neg Control	-3.3791	4.7787	0.0041	
Pos Control vs. IALR1619	-3.9653	5.6078	0.0004	
Pos Control vs. IALR1580	1.3390	1.8936	0.5380	
Neg Control vs. IALR1619	-1.0035	1.4192	0.7474	
Neg Control vs. IALR1580	4.4876	6.3464	<.0001	
IALR1619 vs. IALR1580	4.9457	6.9943	<.0001	

Wilcoxon Scores (Rank Sums) for Variable DryShootWt
Classified by Variable TRT	
TRT	N	Sum of
Scores	Expected
Under H0	Std Dev
Under H0	Mean
Score	
Pos Control	27	834.00	1471.50	140.942124	30.888889	
Neg Control	27	2321.00	1471.50	140.942124	85.962963	
IALR1619	27	1709.50	1471.50	140.942124	63.314815	
IALR1580	27	1021.50	1471.50	140.942124	37.833333	
Average scores were used for ties.	


Kruskal-Wallis Test	
Chi-Square	DF	Pr > ChiSq	
52.3744	3	<.0001	


Pairwise Two-Sided Multiple Comparison Analysis	
Dwass, Steel, Critchlow-Fligner Method	
Variable: DryShootWt	
TRT	Wilcoxon Z	DSCF Value	Pr > DSCF	
Pos Control vs. Neg Control	-5.6487	7.9884	<.0001	
Pos Control vs. IALR1619	-4.3857	6.2023	<.0001	
Pos Control vs. IALR1580	-0.9948	1.4069	0.7524	
Neg Control vs. IALR1619	3.6072	5.1013	0.0018	
Neg Control vs. IALR1580	5.4409	7.6947	<.0001	
IALR1619 vs. IALR1580	3.3390	4.7221	0.0047	

Wilcoxon Scores (Rank Sums) for Variable DryRoot
Classified by Variable TRT	
TRT	N	Sum of
Scores	Expected
Under H0	Std Dev
Under H0	Mean
Score	
Pos Control	25	1400.50	1312.50	131.450805	56.020000	
Neg Control	27	942.50	1417.50	134.867387	34.907407	
IALR1619	27	1901.00	1417.50	134.867387	70.407407	
IALR1580	25	1216.00	1312.50	131.450805	48.640000	
Average scores were used for ties.	


Kruskal-Wallis Test	
Chi-Square	DF	Pr > ChiSq	
19.4494	3	0.0002	


Pairwise Two-Sided Multiple Comparison Analysis	
Dwass, Steel, Critchlow-Fligner Method	
Variable: DryRoot	
TRT	Wilcoxon Z	DSCF Value	Pr > DSCF	
Pos Control vs. Neg Control	2.6010	3.6783	0.0459	
Pos Control vs. IALR1619	-1.8500	2.6163	0.2500	
Pos Control vs. IALR1580	0.9120	1.2898	0.7984	
Neg Control vs. IALR1619	-4.2821	6.0558	0.0001	
Neg Control vs. IALR1580	-1.5661	2.2148	0.3981	
IALR1619 vs. IALR1580	2.4729	3.4972	0.0642	

Wilcoxon Scores (Rank Sums) for Variable DryRoot
Classified by Variable TRT	
TRT	N	Sum of
Scores	Expected
Under H0	Std Dev
Under H0	Mean
Score	
Pos Control	27	1036.50	1471.50	140.934738	38.388889	
Neg Control	27	2122.00	1471.50	140.934738	78.592593	
IALR1619	27	1107.00	1471.50	140.934738	41.000000	
IALR1580	27	1620.50	1471.50	140.934738	60.018519	
Average scores were used for ties.	


Kruskal-Wallis Test	
Chi-Square	DF	Pr > ChiSq	
28.9779	3	<.0001	


Pairwise Two-Sided Multiple Comparison Analysis	
Dwass, Steel, Critchlow-Fligner Method	
Variable: DryRoot	
TRT	Wilcoxon Z	DSCF Value	Pr > DSCF	
Pos Control vs. Neg Control	-4.3512	6.1535	<.0001	
Pos Control vs. IALR1619	-0.3980	0.5629	0.9787	
Pos Control vs. IALR1580	-2.7772	3.9275	0.0281	
Neg Control vs. IALR1619	4.2302	5.9825	0.0001	
Neg Control vs. IALR1580	2.6731	3.7803	0.0377	
IALR1619 vs. IALR1580	-2.4741	3.4990	0.0640	
