# Supplementary material for: Biocontrol potential of endophytic Pseudomonas strain IALR1619 against two Pythium species in cucumber and hydroponic lettuce
Source: PLoS One. 2024 Feb 26;19(2):e0298514. doi: 10.1371/journal.pone.0298514 (PMC10896519; doi:10.1371/journal.pone.0298514)
Supplement: S4 Text — (RTF) [file pone.0298514.s005.rtf]

Obs	Plant_No	Block	Treatment	Shoot_Ht_mm	Shoot_FrWt_g	Root_FrWt_g	Shoot_Dry_g	Root_Dry_g	
1	1	1	1379	229	22.36	11.92	1.85	0.98	
2	2	1	1379	190	13.9	8.1	1.18	0.77	
3	3	1	1379	158	8.27	7.5	0.81	0.71	
4	4	1	1379	199	10.16	7.21	0.97	0.7	
5	5	1	1379	265	34.58	12.76	2.68	1.03	
6	6	1	1379	265	20.93	8.45	1.53	0.77	
7	7	1	1379	245	20.94	10.37	1.63	0.89	
8	8	1	1379	239	20.81	10.2	1.67	0.89	
9	9	1	1379	220	19.9	9.01	1.78	0.84	
10	1	2	1379	227	29.87	9.73	2.25	0.82	
11	2	2	1379	239	33.18	10.27	1.9	0.85	
12	3	2	1379	254	26.31	8.74	2.53	0.78	
13	4	2	1379	275	43.7	11.92	3.37	0.97	
14	5	2	1379	240	22.18	8.14	1.59	0.7	
15	6	2	1379	244	22.61	8.29	1.59	0.72	
16	7	2	1379	260	20.6	7.16	1.4	0.7	
17	8	2	1379	267	25	8.96	1.68	0.76	
18	9	2	1379	250	27.92	10.64	2.16	0.87	
19	1	3	1379	230	18.36	7.35	1.44	0.7	
20	2	3	1379	240	21.06	6.28	1.49	0.66	
21	3	3	1379	240	24.61	7.84	1.88	0.74	
22	4	3	1379	215	14.61	6.59	1.2	0.68	
23	5	3	1379	244	31.6	9.06	2.52	0.83	
24	6	3	1379	271	19.31	7.26	1.35	0.67	
25	7	3	1379	277	27.83	8.01	1.98	0.77	
26	8	3	1379	260	20.46	7.35	1.56	0.74	
27	9	3	1379	243	26.35	10.3	2.2	0.95	
28	1	1	1619	297	59.79	13.62	3.52	0.96	
29	2	1	1619	306	37.4	9.75	2.3	0.78	
30	3	1	1619	279	30.62	9.36	1.87	0.7	
31	4	1	1619	275	42.4	10.72	2.79	0.82	
32	5	1	1619	285	55.79	14.19	3.4	1.05	
33	6	1	1619	272	26.92	7.55	1.58	0.68	
34	7	1	1619	304	49.37	11.79	2.95	0.83	
35	8	1	1619	325	42.62	11.83	2.79	0.86	
36	9	1	1619	302	65.27	15.77	5.09	1.11	
37	1	2	1619	263	31.93	10.23	2.05	0.82	
38	2	2	1619	294	34.28	8.67	2.05	0.76	
39	3	2	1619	290	37.8	9.54	2.32	0.82	
40	4	2	1619	256	35.4	11.58	2.42	0.91	
41	5	2	1619	269	23.3	7.66	1.53	0.74	
42	6	2	1619	273	24.52	7.37	1.52	0.69	
43	7	2	1619	275	26.97	7.31	1.64	0.69	
44	8	2	1619	277	33.4	9.83	2.02	0.79	
45	9	2	1619	220	29.14	9.13	1.84	0.77	
46	1	3	1619	312	55.67	11.31	3.04	0.83	
47	2	3	1619	332	42.31	8.13	2.14	0.7	
48	3	3	1619	355	59.44	10.16	3.07	0.79	
49	4	3	1619	340	51.86	12.18	2.78	0.88	
50	5	3	1619	310	59.29	11.7	3.22	0.89	
51	6	3	1619	302	47.73	10.27	2.57	0.79	
52	7	3	1619	300	41.5	8.87	2.19	0.76	
53	8	3	1619	229	42.565	6.15	2.55	0.58	
54	9	3	1619	302	61.97	12.39	3.7	0.93	
55	1	1	NegCon	305	58.83	15.12	3.76	1.07	
56	2	1	NegCon	314	44.48	9.84	2.85	0.78	
57	3	1	NegCon	304	48.22	10.45	3.02	0.81	
58	4	1	NegCon	258	40.16	14.58	2.9	1	
59	5	1	NegCon	335	71.72	16.41	4.3	1.15	
60	6	1	NegCon	349	56.02	10.46	3.44	0.8	
61	7	1	NegCon	310	62.3	12.9	3.52	0.9	
62	8	1	NegCon	322	56.34	13.44	3.26	0.93	
63	9	1	NegCon	289	56.24	12.98	3.62	0.92	
64	1	2	NegCon	283	28.72	8.41	1.63	0.71	
65	2	2	NegCon	330	63.2	12.02	3.5	0.86	
66	3	2	NegCon	320	53.27	9.2	2.68	0.7	
67	4	2	NegCon	336	87.25	14.61	4.75	0.96	
68	5	2	NegCon	294	50.26	10.95	2.79	0.83	
69	6	2	NegCon	335	42.6	8.21	2.18	0.67	
70	7	2	NegCon	372	34.84	8.08	1.78	0.63	
71	8	2	NegCon	335	40.64	8.83	2.06	0.69	
72	9	2	NegCon	299	64.1	13.68	3.71	0.96	
73	1	3	NegCon	334	59.84	11.2	3.59	0.88	
74	2	3	NegCon	312	52.53	11.29	2.92	0.84	
75	3	3	NegCon	360	61.71	10.97	3.39	0.84	
76	4	3	NegCon	325	35.77	9.14	2.07	0.74	
77	5	3	NegCon	310	60.49	10.87	3.82	0.85	
78	6	3	NegCon	331	58.83	13.75	3.68	0.96	
79	7	3	NegCon	348	66.19	11.97	3.86	0.91	
80	8	3	NegCon	331	44.71	10.43	2.44	0.81	
81	9	3	NegCon	310	58.11	15.43	3.77	1.07	
82	1	1	PosCon	270	20.7	8.05	1.29	0.7	
83	2	1	PosCon	264	24.3	8.44	1.49	0.7	
84	3	1	PosCon	254	18.25	12.3	1.14	0.86	
85	4	1	PosCon	310	48.37	8.08	3	0.68	
86	5	1	PosCon	305	55	12.96	3.3	0.97	
87	6	1	PosCon	300	47.3	9.74	2.68	0.81	
88	7	1	PosCon	298	50.8	12.1	3.03	0.92	
89	8	1	PosCon	258	13.38	5.52	0.79	0.57	
90	9	1	PosCon	285	56.6	13.7	3.62	1.04	
91	1	2	PosCon	260	18.8	7	1.27	0.65	
92	2	2	PosCon	287	36.61	10.68	2.41	0.93	
93	3	2	PosCon	275	27.19	8.41	1.67	0.74	
94	4	2	PosCon	263	31.37	8.96	2.02	0.83	
95	5	2	PosCon	242	11.6	6.73	0.79	0.64	
96	6	2	PosCon	270	24.05	8.34	1.54	0.73	
97	7	2	PosCon	285	23.33	7.87	1.49	0.7	
98	8	2	PosCon	270	29.46	9.35	1.96	0.8	
99	9	2	PosCon	287	37.05	12.39	2.6	0.93	
100	1	3	PosCon	335	58.66	11.31	3.04	0.84	
101	2	3	PosCon	336	60.01	11.28	3.05	0.81	
102	3	3	PosCon	290	28.86	6.46	1.33	0.58	
103	4	3	PosCon	322	48.17	12.22	2.66	0.82	
104	5	3	PosCon	250	20.44	8.19	1.27	0.62	
105	6	3	PosCon	349	43.79	8.08	2.12	0.68	
106	7	3	PosCon	345	39.65	8.31	1.95	0.66	
107	8	3	PosCon	368	52.92	10.37	2.6	0.76	
108	9	3	PosCon	300	49.19	10.9	2.67	0.79	

Class Level Information	
Class	Levels	Values	
Treatment	4	1379 1619 NegCon PosCon	
Block	3	1 2 3	


Data for Analysis of Shoot_FrWt_g Root_Dry_g Shoot_Ht_mm	
Number of Observations Read	108	
Number of Observations Used	108	


Data for Analysis of Shoot_Dry_g	
Number of Observations Read	108	
Number of Observations Used	106	


Note:	Variables in each group are consistent with respect to the presence or absence of missing values.	

Source	DF	Sum of Squares	Mean Square	F Value	Pr > F	
Model	5	14879.45115	2975.89023	21.61	<.0001	
Error	102	14044.27006	137.68892			
Corrected Total	107	28923.72121				


R-Square	Coeff Var	Root MSE	Shoot_FrWt_g Mean	
0.514438	30.10253	11.73409	38.98042	


Source	DF	Type I SS	Mean Square	F Value	Pr > F	
Treatment	3	13328.07312	4442.69104	32.27	<.0001	
Block	2	1551.37803	775.68901	5.63	0.0048	


Source	DF	Type III SS	Mean Square	F Value	Pr > F	
Treatment	3	13328.07312	4442.69104	32.27	<.0001	
Block	2	1551.37803	775.68901	5.63	0.0048	


Source	DF	Sum of Squares	Mean Square	F Value	Pr > F	
Model	5	0.26397130	0.05279426	4.13	0.0019	
Error	102	1.30312037	0.01277569			
Corrected Total	107	1.56709167				


R-Square	Coeff Var	Root MSE	Root_Dry_g Mean	
0.168447	13.95906	0.113030	0.809722	


Source	DF	Type I SS	Mean Square	F Value	Pr > F	
Treatment	3	0.12369907	0.04123302	3.23	0.0256	
Block	2	0.14027222	0.07013611	5.49	0.0054	


Source	DF	Type III SS	Mean Square	F Value	Pr > F	
Treatment	3	0.12369907	0.04123302	3.23	0.0256	
Block	2	0.14027222	0.07013611	5.49	0.0054	


Source	DF	Sum of Squares	Mean Square	F Value	Pr > F	
Model	5	103620.7500	20724.1500	28.75	<.0001	
Error	102	73526.4630	720.8477			
Corrected Total	107	177147.2130				


R-Square	Coeff Var	Root MSE	Shoot_Ht_mm Mean	
0.584941	9.396447	26.84861	285.7315	


Source	DF	Type I SS	Mean Square	F Value	Pr > F	
Treatment	3	89990.62037	29996.87346	41.61	<.0001	
Block	2	13630.12963	6815.06481	9.45	0.0002	


Source	DF	Type III SS	Mean Square	F Value	Pr > F	
Treatment	3	89990.62037	29996.87346	41.61	<.0001	
Block	2	13630.12963	6815.06481	9.45	0.0002	


Treatment	Shoot_FrWt_g LSMEAN	LSMEAN Number	
1379	23.2374074	1	
1619	42.5650000	2	
NegCon	53.9766667	3	
PosCon	36.1425926	4	


Least Squares Means for effect Treatment
Pr > |t| for H0: LSMean(i)=LSMean(j)

Dependent Variable: Shoot_FrWt_g	
i/j	1	2	3	4	
1		<.0001	<.0001	0.0001	
2	<.0001		0.0005	0.0470	
3	<.0001	0.0005		<.0001	
4	0.0001	0.0470	<.0001		


Treatment	Root_Dry_g LSMEAN	LSMEAN Number	
1379	0.79592593	1	
1619	0.81222222	2	
NegCon	0.86185185	3	
PosCon	0.76888889	4	


Least Squares Means for effect Treatment
Pr > |t| for H0: LSMean(i)=LSMean(j)

Dependent Variable: Root_Dry_g	
i/j	1	2	3	4	
1		0.5974	0.0345	0.3815	
2	0.5974		0.1098	0.1620	
3	0.0345	0.1098		0.0032	
4	0.3815	0.1620	0.0032		


Treatment	Shoot_Ht_mm LSMEAN	LSMEAN Number	
1379	240.222222	1	
1619	290.518519	2	
NegCon	320.407407	3	
PosCon	291.777778	4	


Least Squares Means for effect Treatment
Pr > |t| for H0: LSMean(i)=LSMean(j)

Dependent Variable: Shoot_Ht_mm	
i/j	1	2	3	4	
1		<.0001	<.0001	<.0001	
2	<.0001		<.0001	0.8635	
3	<.0001	<.0001		0.0002	
4	<.0001	0.8635	0.0002		


Note:	To ensure overall protection level, only probabilities associated with pre-planned comparisons should be used.	

Source	DF	Sum of Squares	Mean Square	F Value	Pr > F	
Model	5	31.26976536	6.25395307	13.31	<.0001	
Error	100	46.98122803	0.46981228			
Corrected Total	105	78.25099340				


R-Square	Coeff Var	Root MSE	Shoot_Dry_g Mean	
0.399609	28.76417	0.685429	2.382925	


Source	DF	Type I SS	Mean Square	F Value	Pr > F	
Treatment	3	28.33634569	9.44544856	20.10	<.0001	
Block	2	2.93341967	1.46670984	3.12	0.0484	


Source	DF	Type III SS	Mean Square	F Value	Pr > F	
Treatment	3	28.30430411	9.43476804	20.08	<.0001	
Block	2	2.93341967	1.46670984	3.12	0.0484	


Treatment	Shoot_Dry_g LSMEAN	LSMEAN Number	
1379	1.78481481	1	
1619	2.48730640	2	
NegCon	3.15888889	3	
PosCon	2.10296296	4	


Least Squares Means for effect Treatment
Pr > |t| for H0: LSMean(i)=LSMean(j)

Dependent Variable: Shoot_Dry_g	
i/j	1	2	3	4	
1		0.0004	<.0001	0.0912	
2	0.0004		0.0006	0.0461	
3	<.0001	0.0006		<.0001	
4	0.0912	0.0461	<.0001		


Note:	To ensure overall protection level, only probabilities associated with pre-planned comparisons should be used.	
