# Supplementary material for: Biocontrol potential of endophytic Pseudomonas strain IALR1619 against two Pythium species in cucumber and hydroponic lettuce
Source: PLoS One. 2024 Feb 26;19(2):e0298514. doi: 10.1371/journal.pone.0298514 (PMC10896519; doi:10.1371/journal.pone.0298514)
Supplement: S5 Text — (RTF) [file pone.0298514.s006.rtf]

Obs	Plant_Postn	Lettuce_cv	TRT	Shoot_Fr_Wt	Shoot_Dry_Wt	
1	1	Pensacola	Py only	31.69	1.725	
2	3	Pensacola	Py only	27.09	1.479	
3	4	Pensacola	Py only	2.7	0.216	
4	5	Pensacola	IALR1619 + Py	36.4	2.042	
5	6	Pensacola	Py only	34.25	1.964	
6	7	Pensacola	IALR1619 + Py	37.95	1.971	
7	8	Pensacola	IALR1619 + Py	34.95	1.935	
8	9	Pensacola	Py only	27.13	1.603	
9	10	Pensacola	IALR1619 + Py	34.76	1.899	
10	11	Pensacola	IALR1619 + Py	24.49	1.361	
11	13	Pensacola	IALR1619 + Py	30.33	1.66	
12	14	Pensacola	Py only	29.32	1.715	
13	15	Pensacola	Py only	38.08	2.313	
14	16	Pensacola	IALR1619 + Py	23.56	1.318	
15	17	Pensacola	Py only	29.23	1.743	
16	18	Pensacola	Py only	36.36	2.024	
17	19	Pensacola	IALR1619 + Py	27.45	1.555	
18	20	Pensacola	IALR1619 + Py	19.09	1.134	
19	21	Pensacola	Py only	17.55	1.114	
20	22	Pensacola	IALR1619 + Py	7.42	0.445	
21	25	Pensacola	IALR1619 + Py	12.01	0.699	
22	26	Pensacola	Py only	40.94	2.24	
23	27	Pensacola	Py only	34.9	1.96	
24	28	Pensacola	IALR1619 + Py	12.76	0.758	
25	29	Pensacola	Py only	33.72	2.013	
26	30	Pensacola	IALR1619 + Py	22.65	1.296	
27	1	Cristabel	Py only	11.88	0.58	
28	2	Cristabel	IALR1619 + Py	20.5	0.935	
29	3	Cristabel	IALR1619 + Py	32.49	1.449	
30	4	Cristabel	IALR1619 + Py	30.52	1.447	
31	5	Cristabel	IALR1619 + Py	31.08	1.414	
32	6	Cristabel	Py only	10.63	0.575	
33	7	Cristabel	IALR1619 + Py	33.2	1.535	
34	10	Cristabel	Py only	13	0.75	
35	11	Cristabel	Py only	17.63	0.878	
36	12	Cristabel	Py only	23.33	1.17	
37	13	Cristabel	IALR1619 + Py	28.14	1.438	
38	14	Cristabel	IALR1619 + Py	37.73	1.833	
39	15	Cristabel	Py only	14.25	0.747	
40	16	Cristabel	IALR1619 + Py	32.3	1.573	
41	17	Cristabel	Py only	32.6	1.582	
42	18	Cristabel	IALR1619 + Py	33.98	1.614	
43	19	Cristabel	Py only	26.83	1.34	
44	20	Cristabel	Py only	28.12	1.481	
45	21	Cristabel	Py only	24.75	1.222	
46	22	Cristabel	IALR1619 + Py	33.75	1.538	
47	23	Cristabel	IALR1619 + Py	26.42	1.32	
48	24	Cristabel	IALR1619 + Py	27.92	1.341	
49	25	Cristabel	IALR1619 + Py	15.51	0.8	
50	28	Cristabel	Py only	14.69	0.773	
51	29	Cristabel	Py only	12.65	0.677	
52	30	Cristabel	Py only	25.38	1.297	

Class Level Information	
Class	Levels	Values	
TRT	2	IALR1619 + Py Py only	


Number of Observations Read	26	
Number of Observations Used	26	

Source	DF	Sum of Squares	Mean Square	F Value	Pr > F	
Model	1	628.186154	628.186154	13.87	0.0011	
Error	24	1086.597508	45.274896			
Corrected Total	25	1714.783662				


R-Square	Coeff Var	Root MSE	Shoot_Fr_Wt Mean	
0.366336	27.36598	6.728662	24.58769	


Source	DF	Type I SS	Mean Square	F Value	Pr > F	
TRT	1	628.1861538	628.1861538	13.87	0.0011	


Source	DF	Type III SS	Mean Square	F Value	Pr > F	
TRT	1	628.1861538	628.1861538	13.87	0.0011	


Source	DF	Sum of Squares	Mean Square	F Value	Pr > F	
Model	1	1.02604712	1.02604712	10.27	0.0038	
Error	24	2.39868892	0.09994537			
Corrected Total	25	3.42473604				


R-Square	Coeff Var	Root MSE	Shoot_Dry_Wt Mean	
0.299599	26.25340	0.316141	1.204192	


Source	DF	Type I SS	Mean Square	F Value	Pr > F	
TRT	1	1.02604712	1.02604712	10.27	0.0038	


Source	DF	Type III SS	Mean Square	F Value	Pr > F	
TRT	1	1.02604712	1.02604712	10.27	0.0038	


TRT	Shoot_Fr_Wt LSMEAN	H0:LSMean1=LSMean2	
		Pr > |t|	
IALR1619 + Py	29.5030769	0.0011	
Py only	19.6723077		


TRT	Shoot_Dry_Wt LSMEAN	H0:LSMean1=LSMean2	
		Pr > |t|	
IALR1619 + Py	1.40284615	0.0038	
Py only	1.00553846		


Class Level Information	
Class	Levels	Values	
TRT	2	IALR1619 + Py Py only	


Data for Analysis of Shoot_Fr_Wt	
Number of Observations Read	26	
Number of Observations Used	24	


Data for Analysis of Shoot_Dry_Wt	
Number of Observations Read	26	
Number of Observations Used	24	


Note:	Variables in each group are consistent with respect to the presence or absence of missing values.	

Source	DF	Sum of Squares	Mean Square	F Value	Pr > F	
Model	1	210.092873	210.092873	3.03	0.0958	
Error	22	1525.994510	69.363387			
Corrected Total	23	1736.087383				


R-Square	Coeff Var	Root MSE	Shoot_Fr_Wt Mean	
0.121015	30.14194	8.328468	27.63083	


Source	DF	Type I SS	Mean Square	F Value	Pr > F	
TRT	1	210.0928728	210.0928728	3.03	0.0958	


Source	DF	Type III SS	Mean Square	F Value	Pr > F	
TRT	1	210.0928728	210.0928728	3.03	0.0958	


TRT	Shoot_Fr_Wt LSMEAN	H0:LSMean1=LSMean2	
		Pr > |t|	
IALR1619 + Py	24.9092308	0.0958	
Py only	30.8472727		


Source	DF	Sum of Squares	Mean Square	F Value	Pr > F	
Model	1	0.90519032	0.90519032	4.70	0.0412	
Error	22	4.23500831	0.19250038			
Corrected Total	23	5.14019863				


R-Square	Coeff Var	Root MSE	Shoot_Dry_Wt Mean	
0.176100	27.96581	0.438749	1.568875	


Source	DF	Type I SS	Mean Square	F Value	Pr > F	
TRT	1	0.90519032	0.90519032	4.70	0.0412	


Source	DF	Type III SS	Mean Square	F Value	Pr > F	
TRT	1	0.90519032	0.90519032	4.70	0.0412	


TRT	Shoot_Dry_Wt LSMEAN	H0:LSMean1=LSMean2	
		Pr > |t|	
IALR1619 + Py	1.39023077	0.0412	
Py only	1.78000000		
