# Supplementary material for: Biocontrol potential of endophytic Pseudomonas strain IALR1619 against two Pythium species in cucumber and hydroponic lettuce
Source: PLoS One. 2024 Feb 26;19(2):e0298514. doi: 10.1371/journal.pone.0298514 (PMC10896519; doi:10.1371/journal.pone.0298514)
Supplement: Attachment — Submitted filename: Response to Reviewers.docx [file pone.0298514.s007.docx]

**Reviewer #1**: Dear Corresponding Author
I checked your paper and I have some comments to improve it for final publication:

1) Please draw a phylogenetic tree for both Pythium and bacterial isolates that you used in your study.

We thank you for your suggestion. A phylogenetic tree for the isolates in this study has been added to the manuscript. A tree for two Pythium isolates was not prepared since they were not a part of the study. These isolates were verified as *Pythium* using macro and micromorphology and their symptoms on experimented plants resembled *Pythium* blight. There are similar peer reviewed biocontrol publications without phylogenetic trees for pathogens used.

2) You need to have a bar-chart with statistics on growth rate of fungi against bacterial isolates.

As described in the manuscript, we screened >350 endophytes rapidly against a Pythium isolate and selected the 2 best endophytes for testing further in vitro and in greenhouse. First, a dual culture assay was done to ascertain biocontrol ability of these strains in vitro using two Pythium species followed by greenhouse studies. It was not our intention to compare these two using statistics. Therefore, we feel it is not necessary to include a bar chart to show the inhibition of Pythium isolates by bacterial strains in the main body of manuscript. However, a Supplementary Bar-chart figure was added (S1 Fig). In the manuscript, to show statistics, we added standard deviation value after % growth inhibition caused by bacterial endophytes.

3) You need to checke the normality of all of your data. If they are not normal you cannot analyze them with ANOVA.

The normality of data sets was checked using Shapiro-Wilk test using Proc Univariate procedure in SAS. The Dry shoot and root weights of Experiment 3 were not normal. Therefore, a non-parametric test called Kruskal- Wallis was used to analyze these two data sets. Other data sets were either normal or became normal when extreme observations were removed. The manuscript was edited accordingly.

4) How you identify the VOCs? Please add in-vitro photos for that. For examine the VOCs you will need to use two-partitioned-plates or fungal plate on bacterial palte. Please describe it.

We used a fungal plate on a bacterial plate to ascertain if bacterial VOCs have any inhibition on Pythium isolates. This is clearly stated under the heading “Antifungal activity of exudates and VOCs of strain IALR1619” in methods. The assay in our manuscript is named “a two-sealed-bottom-plate assay”. The original manuscript had a photo of the effect of bacterial VOCs on Pythium (see Fig 7 in revised manuscript). The photo shows only the mycelial inhibition and there is no bacterial plate.

5) Why you do not use LC-MS and just go ahead for GC-MS?

We used GC-MS method because VOCs are emitted as gas and not liquid.

Regards

Reviewer #2: The study focuses on the effectiveness of the bacterial endophyte IALR1619 in protecting cucumber seedlings against the soil-borne pathogen P. ultimum. Results show a significant increase in survival rates and long-term effects of the endophyte. Furthermore, experiments on hydroponic lettuce demonstrate improved shoot fresh weight with IALR1619, suggesting its potential as a biocontrol agent against Pythium species in cucumber and hydroponic lettuce systems. I suggest the following changes:

- Keywords should not repeat words from the title.

This journal does not include keywords in its articles. It is for online search only. That is why we also included some words from the title in keywords.

- Unit style is incorrect (it should be given in a form of product, e.g. mg·L-1) .

It was corrected.

- Throughout the text, the authors are incorrectly using the term ‘variety’ instead of ‘cultivar’ and ‘sterilization’ instead of ‘disinfection’.

After some research it was found the lettuce names were cultivars and not varieties. “Variety” was changed to “cultivar” for lettuce. But cucumber Marketmore 76 is a variety. We did not change that. ‘Sterilize’ can be used as a synonym to ‘disinfect’. This word is used only twice. We added ‘surface sterilized’ to avoid any confusion in the first instance. In the second instance, sterilize’ is used to describe disinfestation of grains by autoclaving. We believe it is correct.

- Names of cultivars should be written in ‘’ (‘Cristabel’).

Inverted commas were added to all cultivar names.

- Why are you writing about succulent plants in the Introduction?
Removed words “succulent plants” from the sentence.

- Please, explain the novelty of your study.

The purpose/novelty of the study is mentioned in the introduction. The use of fungicides to manage disease has led to multiple environmental externalities, including resistance development, pollution, and non-target mortality. Growers have limited options as legacy chemistries are withdrawn from the market. Moreover, fungicides are generally labeled for traditional soil-based production, and not for liquid culture systems. Pythium is a common and devastating disease in many crops. Our study was an attempt to find biocontrol agents to control Pythium in hydroponic lettuce greenhouse cucumber. Biocontrol methods are more sustainable and there is a huge market in future. This can be found in the manuscript.

- Reference for the LB medium is missing. Who was the producer?

Manufacturer details added.

- Use mL not ml

ml changed to mL

- Some symbols are use incorrectly, i.e. x instead of ×.

Corrected

- Lux is an outdated unit. Please provide the PPFD value. √

Changed the units.

- Fig. 3 lacks scale bars.

Thank you for highlighting the lack of scale bar in Fig3 (Fig 4 in revised manuscript). This photo was included to show relative sizes of representative plants from different treatments. We don’t have a photo with a ruler, and we don’t think not having a scale bar compromises understanding the growth differences.

- In graphs, name both axes.
We named both axes of Fig 5 graph. However, there are PLOS ONE papers similar to our original graph without a distinct name for X axis (Fig 4 original manuscript) (ex. <https://journals.plos.org/plosone/article?id=10.1371/journal.pone.0286285> ). The figure captions have been changed to make figures more self-explanatory.

- Conclusions are missing.

A concluding statement was added to the end of discussion.

- Reference #2: what year?

The year is 2020 and it is already included

- Minor punctuation corrections are needed.

We have corrected the language to our best.

**Reviewer #3**:

What about the keyword?

The question is not clear. Keywords are added as a requirement of the submission process although they don’t appear in the article.

It is better to added good recommendation in abstract

A summary statement was added to the abstract.

the references that you used are little bit old you should update
References were updated.

Also write your objective clear

The objectives of the study were added at the end of the introduction.

Make sure that all scientific names in the References list are italics.

Corrected.

All tables and Fig must be self-explanatory. This very important because you did not mentioned details about the experiment on the Fig

Where necessary, the Table and Figure captions were added with more content to make them self-explanatory.
