# Supplementary material for: Biocontrol potential of endophytic Pseudomonas strain IALR1619 against two Pythium species in cucumber and hydroponic lettuce
Source: PLoS One. 2024 Feb 26;19(2):e0298514. doi: 10.1371/journal.pone.0298514 (PMC10896519; doi:10.1371/journal.pone.0298514)
Supplement: Attachment — Submitted filename: Response to Reviewers.docx [file pone.0298514.s008.docx]

Reviewer 1

1) This suggestion was discussed and resolved with the Academic editor.

2) A bar chart with statistics on pathogen inhibition by endophytes in vitro have been added as a supporting information figure (S1_Fig)

3) All three endophyte strains (IALR1619, 1580 and 1379) were tested for biocontrol of Pythium species but only IALR1619 gave positive results. That is why the manuscript title mentions only IAR1619 strain. We have included all three bacterial strains in the phylogenetic tree because they were a part of the experiments conducted. Two of them were Pseudomonas species and one was an Enterobacter.

4) Phylogenetic tree was added with more reference sequences from GenBank to corroborate IALR1619’s species identity.

5) The GC-MS analysis resulted only in one tall peak, and this was identified as 1-undecene. This is mentioned in the results section. There may be other VOCs produced by IALR1619 but not detected due to their relatively weak signals in the presence of 1-undecene. Therefore, further investigations are needed in future to identify additional fungicidal or fungistatic VOCs. This is also mentioned in the discussion.

Reviewer 2

We agree disinfestation and sterilization are not the same and regret the miscommunication. We sterilized the grains by autoclaving in order to prepare the grain inoculum and not for germination.
